# PGAM1 regulation of ASS1 contributes to the progression of breast cancer through the cAMP/AMPK/CEBPB pathway

**DOI:** 10.1002/1878-0261.13259

**Published:** 2022-06-27

**Authors:** Min Liu, Runmei Li, Min Wang, Ting Liu, Qiuru Zhou, Dong Zhang, Jian Wang, Meng Shen, Xiubao Ren, Qian Sun

**Affiliations:** ^1^ Department of Immunology, National Clinical Research Center for Cancer, Key Laboratory of Cancer Prevention and Therapy, Key Laboratory of Cancer Immunology and Biotherapy, Tianjin's Clinical Research Center for Cancer Tianjin Medical University Cancer Institute and Hospital China

**Keywords:** *AMPK*, *ASS1*, breast cancer, *CEBPB*, *PGAM1*

## Abstract

Phosphoglycerate mutase 1 (*PGAM1*) is a crucial glycolytic enzyme, and its expression status has been confirmed to be associated with tumor progression and metastasis. However, the precise role and other biological functions of *PGAM1* remain unclear. Here, we report that *PGAM1* expression is upregulated and related to poor prognosis in patients with breast cancer (BC). Functional experiments showed that knockdown of *PGAM1* could suppress the proliferation, invasion, migration, and epithelial–mesenchymal transition of BC cells. Through RNA sequencing, we found that argininosuccinate synthase 1 (*ASS1*) expression was markedly upregulated in BC cells following *PGAM1* knockdown, and it is required to suppress the malignant biological behavior of BC cells. Importantly, we demonstrated that *PGAM1* negatively regulates *ASS1* expression through the *cAMP/AMPK/CEBPB* axis. *In vivo* experiments further validated that *PGAM1* promoted tumor growth in BC by altering *ASS1* expression. Finally, immunohistochemical analysis showed that downregulated ASS1 levels were associated with *PGAM1* expression and poor prognosis in patients with BC. Our study provides new insight into the regulatory mechanism of *PGAM1*‐mediated BC progression that might shed new light on potential targets and combination therapeutic strategies for BC treatment.

## Graphical abstract

Knockdown of phosphoglycerate mutase 1 (PGAM1) could reconstruct the expression of ASS1 in breast cancer (BC) cells, resulting in a decrease in tumor growth and exerting antitumor effects via cAMP/AMPK/CEBPB axis. These findings constructed a new theoretical background for PGAM1‐mediated tumor progression, and the elements in this molecular network could be the target of BC treatment.

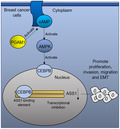

**Abbreviations**
AICAR: aminoimidazole‐4‐carboxamide ribonucleoside
AMPK: AMP‐activated protein kinase
ASS1: argininosuccinate synthase 1
BC: breast cancer
cAMP: cyclic adenosine monophosphate
CEBPB: CCAAT‐enhancer‐binding protein beta
co‐IP: co‐immunoprecipitation
DEGs: differentially expressed genes
DFS: disease‐free survival
EMT: epithelial–mesenchymal transition
ER: estrogen receptor
FOXO: Forkhead box O
H&E: hematoxylin and eosin
HCC: hepatocellular carcinoma
HER2: human epidermal growth factor receptor 2
HIF‐1: hypoxia‐inducible factor 1
IHC: immunohistochemistry
KEGG: Kyoto Encyclopedia of Genes and Genomes
LN: lymph node
mTOR: mammalian target of rapamycin
NSCLC: non‐small cell lung cancer
OS: overall survival
PGAM1: phosphoglycerate mutase 1
PR: progesterone receptor
RTCA: real‐time cell analysis
RT‐PCR: quantitative reverse transcription‐polymerase chain reaction
S1P: sphingosine 1‐phosphate
shPGAM1: *PGAM1*‐knockdown
shScr: *PGAM1*‐negative control
siASS1: ASS1‐siRNA
siCEBPB: CEBPB‐siRNA
siNC: Negative control‐siRNA
siPGAM1: PGAM1‐siRNA
TCGA: Cancer Genome Atlas
TCIH: Tianjin Medical University Cancer Institute and Hospital
*TIF‐1A*
: transcription initiation factor 1A
TNM: tumor‐necrosis‐metastasis

## 1 Introduction

Breast cancer (BC) is one of the most common causes of cancer‐related deaths in women in developing countries [1]. Globally, approximately 2 million cases were registered in 2018; this number is estimated to add up to more than 3 million by 2040. However, the high mortality rate is mainly associated with metastatic cancers. The 5‐year survival rate for BC patients is 99% when the tumor is localized, and drops to 27% due to metastasis [2]. Therefore, elaborating the potential molecular mechanisms of tumor progression and metastasis is the key to BC therapy.

Phosphoglycerate mutase 1 (PGAM1) is a critical glycolytic enzyme involved in tumor progression and metastasis [3]. Accumulating evidence has demonstrated that PGAM1 is upregulated in some types of tumors, including gliomas [4], bladder cancer [5], non‐small cell lung cancer (NSCLC) [6], and renal clear cell carcinoma [7]. We have previously reported that overexpression of *PGAM1* is critical for the oncogenic mammalian target of rapamycin (mTOR)‐mediated Warburg effect [8]. The blockade of PGAM1 alleviates the mTOR‐dependent glycolysis process and tumorigenesis [8]. A recent study revealed a pivotal non‐enzymatic function of PGAM1: in glioma, the overexpressed PGAM1 binds to the cytoplasmic phosphatase wild‐type p53‐induced phosphatase 1 and prevents its nuclear translocation and relevant dephosphorylated reaction, which decreases the curative effect of irradiation and chemotherapy [9]. Taken together, these findings suggested the importance of PGAM1 as a therapeutic target. It is imperative to dissect the molecular mechanisms of PGAM1 in tumorigenesis to enable the development of new PGAM1 inhibitors for tumor combination therapy [10].

Argininosuccinate synthase 1 (ASS1) is a key enzyme of arginine metabolism. Downregulation of ASS1 has been reported in melanoma, renal cell carcinoma, mesothelioma, and pancreatic cancer [11, 12, 13]. The lower expression of ASS1 in tumor samples is related to the poor prognosis of patients with hepatocellular carcinoma (HCC) [14]. However, without ASS1, tumor cells are unable to synthesize arginine from citrulline and are dependent on exogenous arginine. Thus, arginine depletion treatment named ‘arginine starvation’ was utilized in treating individuals with ASS1‐deficient tumors [15, 16]. Nevertheless, most treatment strategies for ASS1‐deficient tumors neglect the tumor‐suppressive effect of ASS1. Actually, the method of reconstructing the activity or expression of ASS1 in cancer cells is particularly important for tumor therapy [17]. In BC, Zou et al. [17] discovered that spinosyn A and its derivatives significantly inhibit tumor growth by activation of ASS1. In HCC, treatment with decitabine could increase ASS1 expression, thereby facilitating the robust therapeutic activity of combined decitabine and anti‐HCC therapies like cisplatin [18]. And in renal cell carcinoma, lncRNA 00312 can also inhibit tumor cell proliferation and invasion *in vitro* by suppressing miR‐34a‐5p and overexpressing ASS1 [19].

It is well known that cyclic adenosine monophosphate (cAMP) is one of the most frequent secondary messengers, participating in many biological effects, including metabolism, cell proliferation, and differentiation [20]. cAMP signaling was capable of promoting AMP‐activated protein kinase (AMPK) activity to dominate the activity of downstream target in many types of cells [21, 22]. However, as an energy sensor, the AMPK pathway activates or mediates many effector molecules to promote or suppress the tumor growth, indicating the double‐faced behaviors of AMPK on tumor development [23, 24]. CCAAT‐enhancer‐binding protein beta (*CEBPB*), transcription initiation factor 1A (*TIF‐1A*), hypoxia‐inducible factor 1 (*HIF‐1*), and Forkhead box O (*FOXO*) are common transcription factors downstream of AMPK pathway [25, 26].

In this study, we revealed a new mechanism whereby the tumor cells regain the expression of ASS1 by the knockdown of *PGAM1*. PGAM1 was upregulated in BC tissues and correlated with worse patient prognosis. *In vitro* and *In vivo* experiments validated that ASS1 is required for controlling the PGAM1‐mediated malignant biological behaviors of BC. Furthermore, PGAM1 negatively regulated ASS1 expression through cAMP/AMPK/CEBPB axis. Immunohistochemical analysis showed that downregulated ASS1 levels were associated with worse prognosis and upregulated PGAM1 expression in patients with BC. Our results demonstrate a better understanding of PGAM1‐mediated molecular network in tumor formation, and therefore, this cascade might be targeted for BC therapy.

## 2 Methods

### 2.1 Animals, cell lines

Five‐week‐old female BALB/C nude mice, were obtained from Sibeifu Corporation (Beijing, China). Animal care was conducted in the pathogen‐free barrier facility at Tianjin Medical University Cancer Institute and Hospital (TCIH) as required by the guidelines of the Animal Care and Use Committee. All animal experiments were approved by the Laboratory Animal Ethics Committee at Tianjin Medical University Cancer Hospital and Institute (No. LLSP2019‐040). BC cell lines, were mycoplasma‐free cells and bought from the American Type Culture Collection (Manassas, VA, USA), identified using short tandem repeat profiling. The catalog numbers and concentrations of reagents are provided in Table S1.

### 2.2 Online database analysis

We performed the prognostic value and differential expression analysis of *PGAM1* in BC using University of ALabama at Birmingham CANcer data analysis (UALCAN). The transcription factor binding sites of *ASS1* were analyzed by GeneCards (The Human Gene Database). The scores of *ASS1* and transcription factor binding were evaluated using Cistrome Data Browser.

### 2.3 Patient samples and immunohistochemical staining

All BC tumor tissues were provided by TCIH. This research was authorized by the Ethics Committee of TCIH. The study methodologies conformed to the standards set by the Declaration of Helsinki. The experiments were undertaken with the understanding and written consent of each subject. Patients with multiple primary cancers, autoimmune diseases, infectious diseases, or neoadjuvant therapy were excluded from the study. Immunohistochemical staining was performed as previously described [8]. The pathologists, blinded to the group designations, evaluated the results. According to the overall staining intensity, the pathologists classified the samples into negative, 0; weak, 1; moderate, 2; and strong, 3. The staining frequency was 0–100%. PGAM1 and ASS1 expression levels were assessed by multiplying staining intensity and frequency scores. The catalog numbers and concentrations of the reagents are listed in Table S1.

### 2.4 Lentiviral transduction and small interfering RNA (siRNA) transfection

#### 2.4.1 Lentiviral transduction

Lentiviruses of *PGAM1*‐knockdown (shPGAM1) and *PGAM1*‐negative control (shScr) were purchased from GeneChem (Shanghai, China). The sequences of shScr and shPGAM1 are provided in Table S2. shScr and shPGAM1 lentiviruses were transduced into BC cells using HiltransG P Reagent (GeneChem). After 72 h, stable cell lines were selected with puromycin, and the efficiency was evaluated by western blotting.

#### 2.4.2 siRNA transfection

Negative control‐siRNA (siNC), *ASS1*‐siRNA (siASS1), *PGAM1*‐siRNA (siPGAM1), and *CEBPB*‐siRNA (siCEBPB) were obtained from GenePharma (Shanghai, China). The sequences of the siRNAs are provided in Table S2. The cells were transfected with siRNAs at an optimal concentration of 20 nm using RNAiMAX transfection reagent (Invitrogen, Carlsbad, CA, USA) and measured after 48 h for subsequent analysis and functional experiments.

### 2.5 Western blotting

Cells were lysed in protein lysis buffer and boiled for analysis. The obtained proteins were subjected to electrophoresis, membrane transfer and blocking, and the primary antibodies (anti‐GAPDH, anti‐β‐Tubulin, anti‐PGAM1, anti‐ASS1, anti‐Slug, anti‐Snail, anti‐E‐cadherin, anti‐Vimentin, anti‐N‐cadherin, anti‐AMPK, anti‐pAMPK, anti‐CEBPB, and anti‐pCEBPB) were incubated overnight at 4 °C. After that, these membranes were then incubated with secondary antibody and visualized by the Image Studio software (Lincoln, NE, USA). The catalog numbers and concentrations of the reagents are listed in Table S1.

### 2.6 Cell proliferation, invasion, and wound healing assays *in vitro*

For the cell proliferation assay, 50 μL RPMI 1640 medium was added to a real‐time cell analysis (RTCA) resistor plate (Agilent, San Diego, CA, USA), and the plate was placed on a super clean bench for 30 min to detect the base value. Then, 5 × 10^3^ BC cells per well in a 100‐μL suspension were seeded into the plate and measured automatically every 15 min using the xCELLigence RTCA DPlus system (Agilent).

For the invasion assay, 2 × 10^4^ BC cells were cultured in 8‐μm transwell upper chambers precoated with Matrigel (BD Biosciences, Fanklin Lakes, NJ, USA). The lower chamber was added 500 μL of RPMI 1640 medium containing 10% fetal bovine serum. After 36 h (MDA‐MB‐231) or 52 h (MCF‐7) at 37 °C, the invasion abilities were evaluated by counting the number of invading cells after fixing and staining with crystal violet.

For the wound healing assays, 5 × 10^5^ BC cells per well were added in 6‐well plates. After the cells reached confluence, sterile pipettes were used to scratch a straight line, and then the cell debris were washed twice with PBS. Finally, these cells were cultured in RPMI 1640 medium and recorded by inverted microscopes (Olympus, Tokyo, Japan) at 12, 24, and 48 h. The scratch areas were measured using ImageJ (Bethesda, MA, USA) software.

### 2.7 RNA‐sequence analysis

Total RNA was extracted from 12 samples, six negative control samples (three shScr‐MDA‐MB‐231 and three shScr‐MCF‐7), and six knockdown samples (three shPGAM1‐MDA‐MB‐231 and three shPGAM1‐MCF‐7), as required by the standard TRIzol protocol. cDNA library construction and sequencing were conducted by Gene Denovo Biotechnology Co., Ltd. (Guangzhou, China). Transcriptome data analyses were analyzed by an online platform (Omicsmart, Guangzhou, China).

### 2.8 Quantitative reverse transcription‐polymerase chain reaction (RT‐PCR)

The RT‐PCR experiment was conducted as previously described [8]. The primers used are provided in Table S3.

### 2.9 Co‐immunoprecipitation (Co‐IP)

Cells in culture dishes were lysed with IP buffer and phenylmethanesulfonyl fluoride in a ratio of 100 : 1. The extract was obtained from the supernatant after centrifugation of the lysates. Sixty microlitre of supernatant was kept for input, and the rest of the extract was divided into equal volumes to be incubated with anti‐IgG, anti‐PGAM1, or anti‐ASS1 antibodies overnight at 4 °C. Then, the Dynabeads (Invitrogen) were washed with IP buffer, added to the extract, and the mixtures were incubated for 2 h. The immunoprecipitations were washed with IP buffer and resuspended in the loading buffer for being boiled. Finally, Dynabeads were removed from the loading buffer, and the rest of the loading buffer was used for western blotting.

### 2.10 Enzyme‐linked immunosorbent assay

cAMP ELISA kit (Bioswamp, Wuhan, China) was used to detect the levels of cAMP per the manufacturer's instructions.

### 2.11 Chromatin immunoprecipitation

The *ASS1* promoter‐binding transcription factors were forecasted by JASPAR database. SimpleChIP® Plus Enzymatic Chromatin IP Kit (CST, Danvers, MA, USA) ChIP assays were used for identification. BC cells were 90% confluent in 15 cm plates with 1% formaldehyde added. Then, cells were incubated with Micrococcal Nuclease (CST) and DNA was sheared by sonication. Transferred supernatants were immunoprecipitated with anti‐CEBPB antibodies (Proteintech, Wuhan, China) and corresponding rabbit‐IgG (CST) at 4 °C overnight. Finally, the DNA extracted from the immunoprecipitations was used to amplify the primers of *ASS1* promoter 838–848 for RT‐PCR verification: 5′‐CTTCATGTACCTGAACGAAGTC‐3′ and 5′‐TCGATGTCTAAATGAGCATGGT‐3′.

### 2.12 Tumor xenograft models

MDA‐MB‐231 cells were transduced with the shPGAM1 or shScr lentiviral vectors. After that, shPGAM1‐MDA‐MB‐231 cells were infected with siNC or siASS1. Next, all BALB/c nude mice were randomly assigned to four groups: shScr, shPGAM1, shPGAM1+siNC, and shPGAM1+siASS1. The MDA‐MB‐231 cells, treated as aforementioned, were injected subcutaneously into the groins of mice in the four groups (5 × 10^6^ cells/100 μL). Tumor volumes were measured once a week and evaluated using the formula, longest axis × (shortest axis)^2^/2. The tumor tissues were weighed, photographed, and used for hematoxylin and eosin (H&E) staining and immunohistochemical analysis of the Ki67 protein (Abcam, Cambridge, UK) after 4 weeks. The catalog numbers and concentrations of the reagents are listed in Table S1.

### 2.13 Statistical analysis

Data was analyzed by *t*‐test, or one‐way ANOVA, using GraphPad (San Diego, CA, USA), and was shown as mean ± SD. A two‐tailed *P* value < 0.05 was considered statistically significant. The relationship between the clinicopathological features of patients and the expression levels of PGAM1 and ASS1 was assessed by χ^2^ test. The correlations between PGAM1 and ASS1 were detected using Spearman's correlation. Kaplan–Meier analysis was conducted with ‘low’ or ‘high’ groups based on the median of the expression levels of PGAM1 and ASS1. In univariate and multivariate analyses, Cox regression was performed to quantify the risk ratio of BC death.

## 3 Results

### 3.1 High expression of *PGAM1* in BC is significantly correlated with poor prognosis

**Fig. 1:**
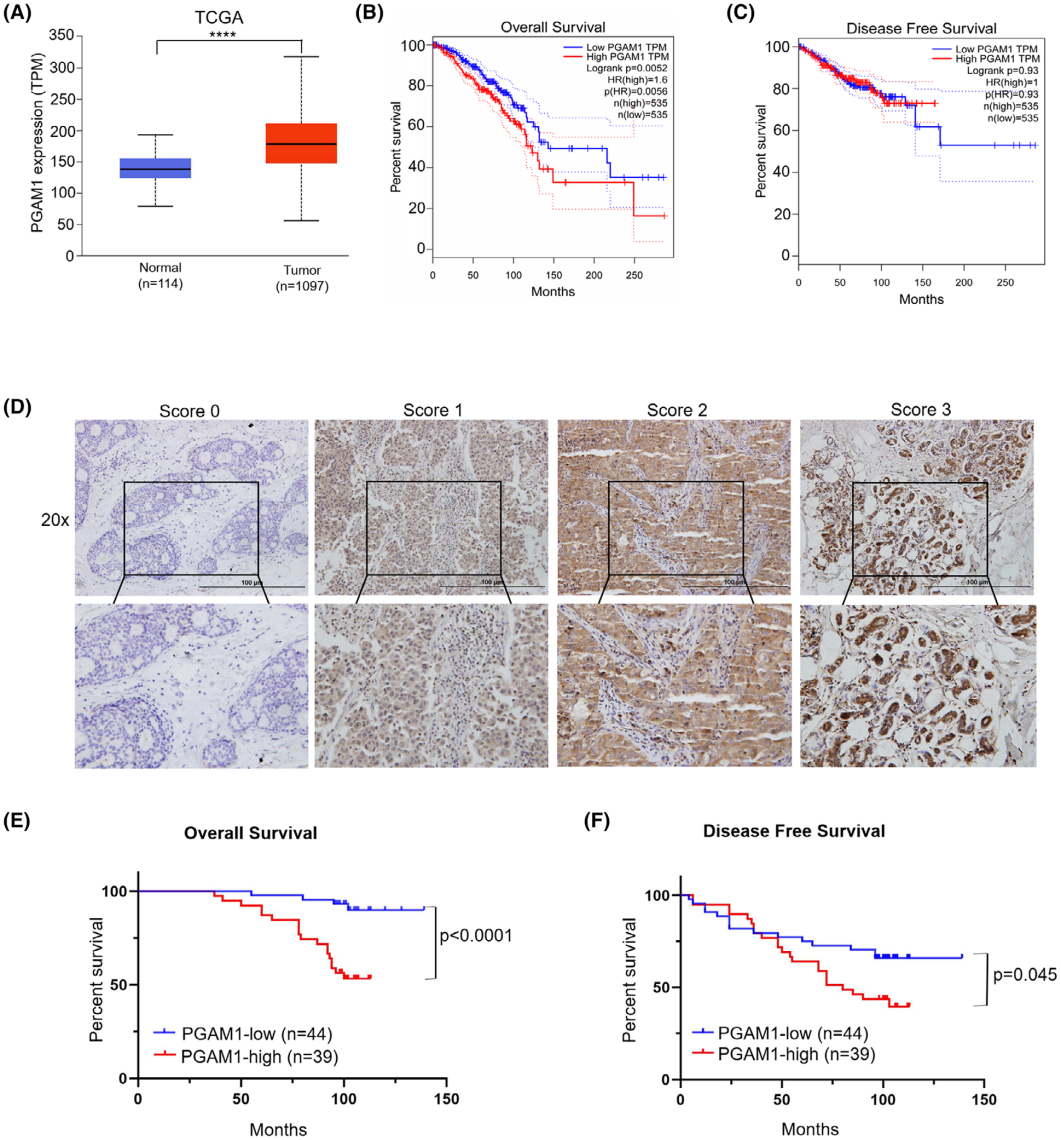
High expression of PGAM1 in BC significantly correlated with poor prognosis. (A) The mRNA expression levels of PGAM1 were analyzed in BC or control group from TCGA databases. (B) OS rate and (C) DFS rate analysis of the BC patients from TCGA databases. (D) Representative images of PGAM1 with different IHC staining scores (*n* = 83, scale bar: 100 μm). (E) Kaplan–Meier analysis of the OS and (F) DFS in BC patients (*n* = 83). Data was analyzed by *t*‐test (A) or Kaplan–Meier method (E, F), and was shown as mean ± SD. ****P < 0.0001.

To explore the role of PGAM1 in BC, we analyzed the expression levels of PGAM1 in tumor and non‐tumor control tissues from the Cancer Genome Atlas (TCGA) database. We found that PGAM1 was upregulated in tumor samples compared to the control group (Fig. 1A). We then determined the prognostic effect of PGAM1 in the TCGA cohort. Kaplan–Meier analysis demonstrated that the overall survival (OS) of patients with high PGAM1 expression was significantly worse than that of patients with low PGAM1 expression (Fig. 1B), while disease‐free survival (DFS) (Fig. 1C) was not statistically significant. Next, we analyzed tumor tissue samples from 83 BC patients to further assess the effects of PGAM1. Representative images of immunohistochemical staining for PGAM1 are shown in Fig. 1D. Kaplan–Meier analysis revealed that high expression of PGAM1 was significantly related to poor OS (Fig. 1E) and DFS (Fig. 1F). Besides, PGAM1 has no obvious correlation with the clinical pathological characteristics of patients (Table 1) including age; lymph node (LN) metastasis; tumor‐necrosis‐metastasis (TNM) stage; and estrogen receptor (ER), progesterone receptor (PR), and human epidermal growth factor receptor 2 (HER2) statuses. Therefore, these findings indicate that highly expressed PGAM1 positively correlates with worse prognosis, and may act as a diagnostic and prognostic biomarker for BC patients.

**Table 1:** Correlation analysis between PGAM1, ASS1, and clinicopathologic features of the patients with breast cancer.

Variables	All cases	PGAM1 (%)		*P* value	ASS1 (%)		*P* value
		Low	High		Low	High	
Age							
< 50 years	42	26 (61.9)	16 (38.1)	0.100	23 (54.8)	19 (45.2)	0.903
≥ 50 years	41	18 (43.9)	23 (56.1)		23 (56.1)	18 (43.9)	
LN metastasis							
Yes	41	22 (53.7)	19 (46.3)	0.907	22 (53.7)	19 (46.3)	0.75
No	42	22 (52.4)	20 (47.6)		24 (57.1)	18 (42.9)	
TNM stage							
I	27	15 (55.6)	12 (44.4)	0.536	14 (51.9)	13 (48.1)	0.849
II	41	23 (56.1)	18 (43.9)		24 (58.5)	17 (41.5)	
III	15	6 (40)	9 (60)		8 (53.3)	7 (46.7)	
ER							
Positive	56	30 (53.6)	26 (46.4)	0.883	31 (55.4)	25 (44.6)	0.986
Negative	27	14 (51.9)	13 (48.1)		15 (55.6)	12 (44.4)	
PR							
Positive	46	26 (56.5)	20 (43.5)	0.475	28 (60.9)	18 (39.1)	0.266
Negative	37	18 (48.6)	19 (51.4)		18 (48.6)	19 (51.4)	
HER2							
Positive	42	21 (50)	21 (50)	0.578	23 (54.8)	19 (45.2)	0.903
Negative	41	23 (56.1)	18 (43.9)		23 (56.1)	18 (43.9)	

### 3.2 Knockdown of *PGAM1* inhibits BC cell proliferation, invasion, migration, and epithelial–mesenchymal transition process *in vitro*

**Fig. 2:**
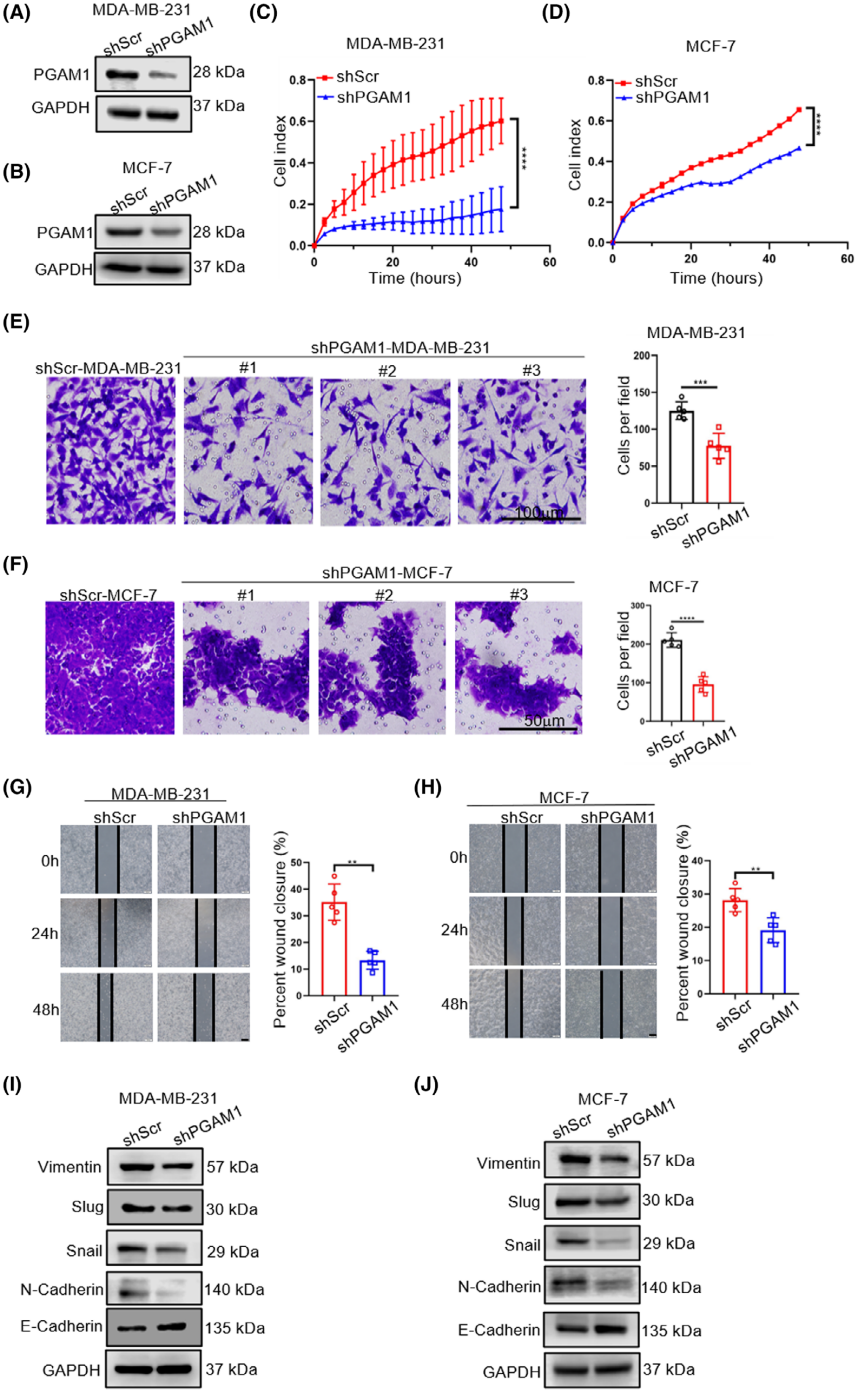
Knockdown of *PGAM1* inhibits BC cell proliferation, invasion, migration, and EMT process *in vitro*. (A, B) *PGAM1* knockdown efficiencies following lentivirus transduction detected by western blot in BC cell lines (*n* = 3). (C, D) Proliferative abilities of shScr‐ and shPGAM1‐BC cell lines detected by cell proliferation assays (*n* = 3). (E, F) Representative images of shScr‐ and shPGAM1‐BC cell lines from transwell invasion assays [*n* = 5, scale bar: 100 μm (E), 50 μm (F)]. (G, H) Migration abilities of shScr‐ and shPGAM1‐BC cell lines detected by wound healing assay (*n* = 5). Scale bar: 100 μm. (I, J) The protein levels of E‐cadherin, N‐cadherin, Vimentin, Snail, and Slug in BC cells detected by western blot (*n* = 3). Data was analyzed by ANOVA (C, D) or *t*‐test (E–H), and was shown as mean ± SD. **P < 0.01, and ***P < 0.001, ****P < 0.0001.

To further determine the biological functions of PGAM1 in BC cells, we used lentiviral vectors to knock down the expression of *PGAM1* in MDA‐MB‐231 and MCF‐7 cells (Fig. 2A,B). Continuous monitoring of cell proliferation revealed that *PGAM1* knockdown inhibited the proliferation of BC cells (Fig. 2C,D). Next, we detected the impact of PGAM1 on BC invasion and migration through transwell and wound‐healing assays, respectively. Compared with shScr groups, the invasion (Fig. 2E,F) and migration (Fig. 2G,H) capacities of the shPGAM1 groups were distinctly decreased. It is well known that epithelial–mesenchymal transition (EMT) closely correlates with tumor invasion and metastasis [27]. In the EMT process, tumor cells acquire mobility through changes from an epithelial‐like to a mesenchymal‐like phenotype [27]. Thus, we also detected the protein levels of EMT‐related markers, including an epithelial marker (E‐cadherin) and mesenchymal markers (N‐cadherin, Vimentin, Snail, and Slug). The western blot assay showed that silence of *PGAM1* increased the levels of the epithelial marker and significantly decreased the levels of mesenchymal markers. Moreover, we repeated the above experiments using another siRNA sequence to silence *PGAM1* in BC cells. The results showed that these observations were recapitulated in additional *siPGAM1* sequence (Fig. S1), strongly suggesting that the knockdown of *PGAM1* expression suppresses the malignant biological behavior of BC cells, and that PGAM1 is important for BC progression and metastasis.

### 3.3 *ASS1* is identified to be upregulated in *PGAM1* knockdown BC cells

**Fig. 3:**
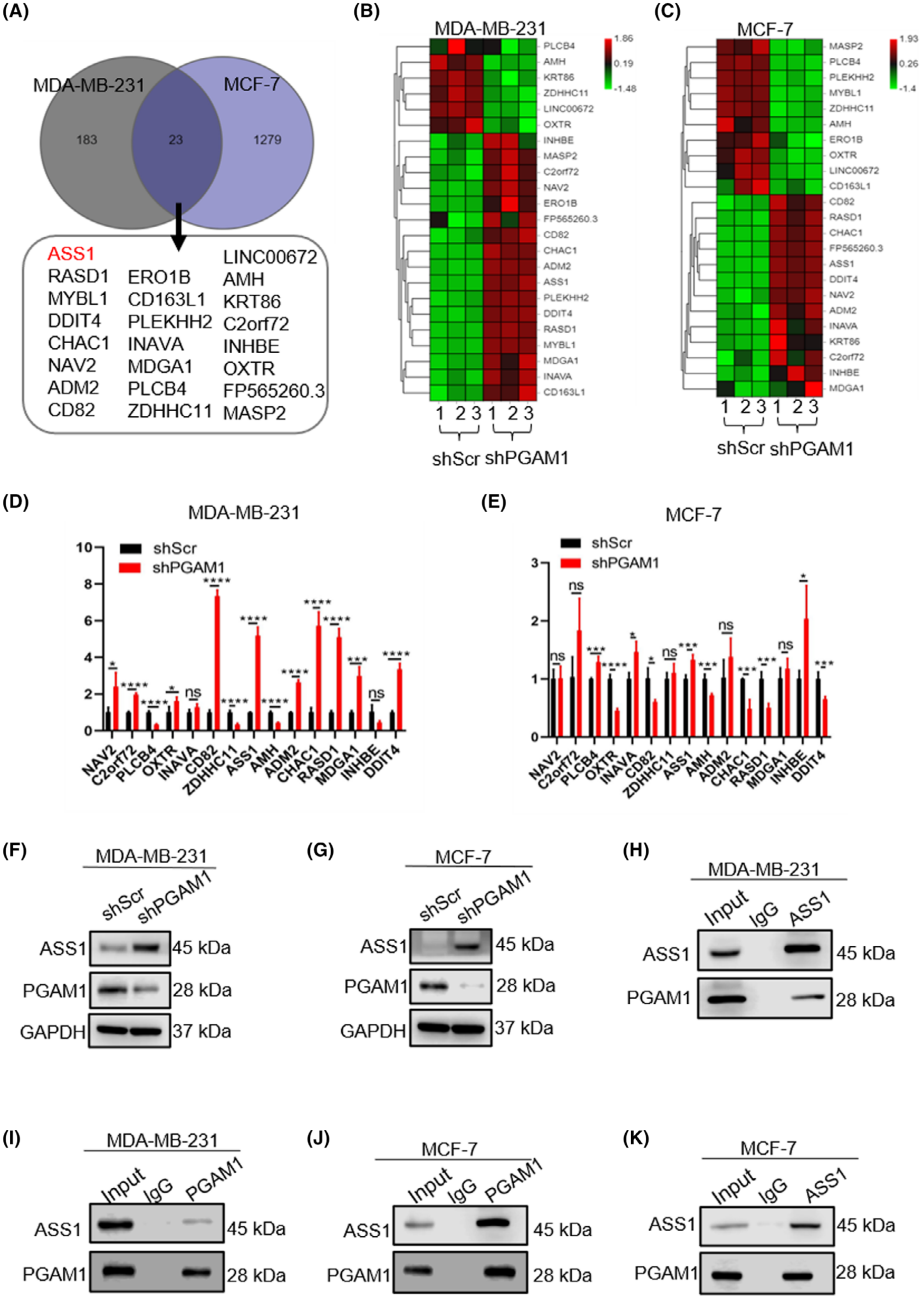
*ASS1* is identified to be upregulated in *PGAM1* knockdown BC cells. (A) Venn diagram showing the DEGs between the shScr and shPGAM1 groups (*n* = 3). (B, C) The specific changes of DEGs in shScr and the shPGAM1 groups are shown by heat maps (*n* = 3). (D, E) The mRNA levels of DEGs in the shScr and shPGAM1 groups detected by RT‐PCR (*n* = 3). (F, G) The protein levels of PGAM1 and ASS1 measured by western blot (*n* = 3). (H–K) The interaction between PGAM1 and ASS1 demonstrated through co‐IP assay (*n* = 3). (Data was analyzed by *t*‐test, and was shown as the mean ± SD. ns, no statistically significant; *P < 0.05, ***P < 0.001, and ****P < 0.0001.).

To identify the crucial downstream substrates of PGAM1 involved in BC progression, we used RNA‐Sequencing to compare mRNA expression profiles between three paired shScr and shPGAM1 BC cell lines. Transcriptome data analysis showed that there were 183 differentially expressed genes (DEGs) in MDA‐MB‐231 cells between the shScr and shPGAM1 groups, and 1279 DEGs in MCF‐7 cells between the two groups (Fig. 3A). Among the 23 overlapping genes between MDA‐MB‐231 (Fig. 3B) and MCF‐7 cells (Fig. 3C), we analyzed genes that showed co‐upregulated or co‐downregulated expression patterns to verify their mRNA levels. RT‐PCR results confirmed that only the changes in anti‐Mullerian hormone and *ASS1* levels were consistent between the two cell lines (Fig. 3D,E). Subsequently, we focused on the highly ranked *ASS1*. We first validated that the protein levels of ASS1 were consistent with the mRNA levels. Compared with the shScr group, ASS1 was upregulated in the shPGAM1‐BC cell lines (Fig. 3F,G, Fig. S1), suggesting that the expression of ASS1 was regulated by PGAM1. We proposed that ASS1 could be an important downstream substrate of PGAM1. ASS1 is known as the rate‐limiting enzyme in arginine metabolism [28]. Notably, recent studies deepened our insights into the role of ASS1 as a novel tumor suppressive character [17, 29]. Thus, we were urged to explore the biological association of PGAM1 and ASS1. We first demonstrated the interaction via the co‐IP assay. IP of endogenous *ASS1* with anti‐ASS1 antibody pulled down endogenous PGAM1 in MDA‐MB‐231 cells (Fig. 3H). Meanwhile, endogenous ASS1 was pulled down by endogenous PGAM1 with anti‐PGAM1 (Fig. 3I). This phenomenon was recapitulated in another human BC cell line MCF‐7 (Fig. 3J,K). Accordingly, these results effectively confirmed the interaction between PGAM1 and ASS1, and *ASS1* was considered a potential target of *PGAM1* in BC regulation.

### 3.4 *ASS1* is required for *PGAM1* ‐mediated BC proliferation, invasion, migration, and EMT process *in vitro*

**Fig. 4:**
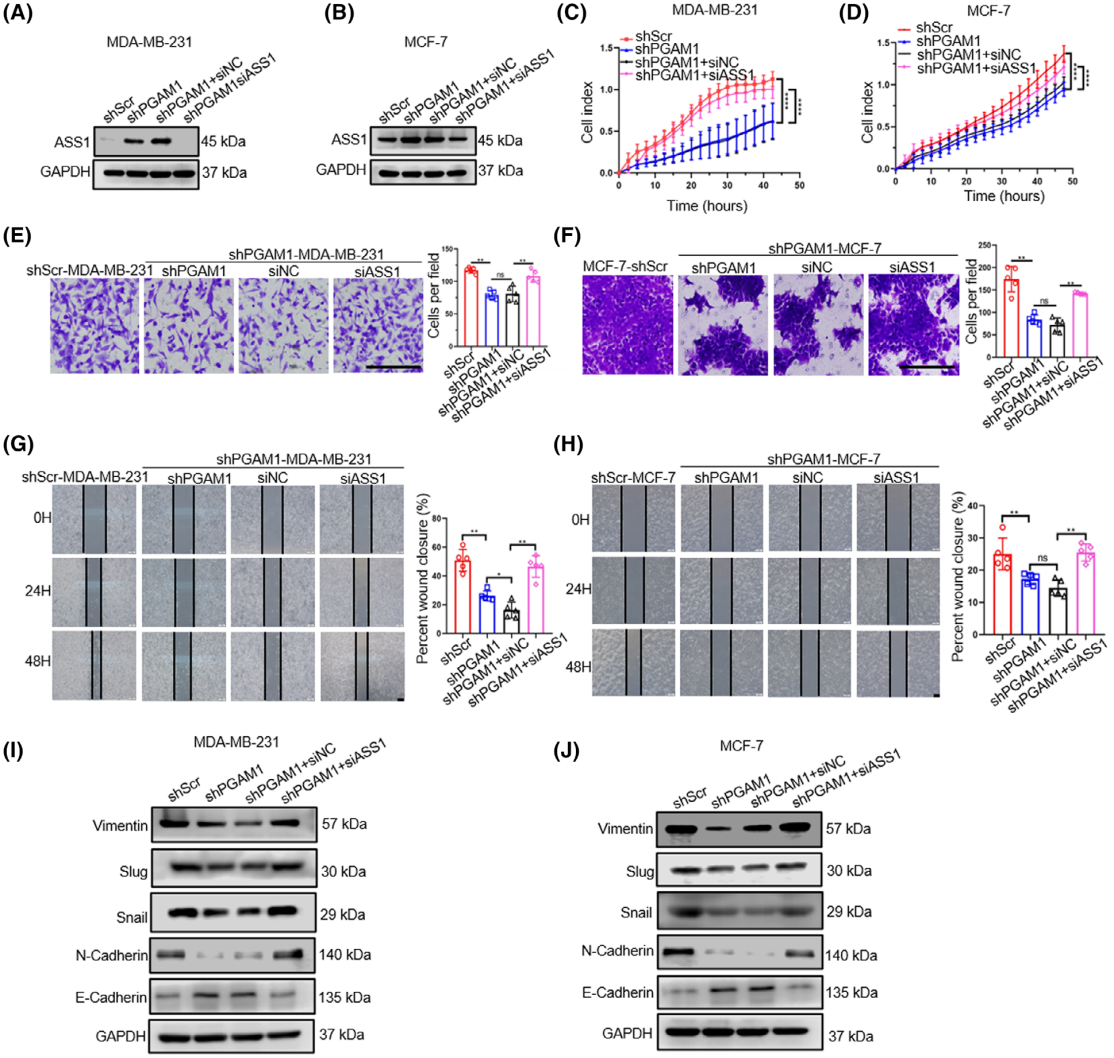
ASS1 is required for suppressing PGAM1‐mediated BC proliferation, invasion, migration, and EMT process *in vitro*. (A, B) *ASS1* knockdown efficiencies detected by western blot (*n* = 3). (C, D) Proliferative abilities of *ASS1*‐knockdown *shPGAM1*‐BC cell lines detected by cell proliferation assays (*n* = 3). (E, F) Invasion and (G, H) migration abilities of *ASS1*‐knockdown *shPGAM1*‐BC cell lines detected by transwell invasion and wound healing assays, respectively (*n* = 5, scale bar: 100 μm (E, G, H), 50 μm (F)). (I, J) Protein levels of E‐cadherin, N‐cadherin, vimentin, Snail, and Slug in BC cells detected by western blot (*n* = 3). Data was analyzed by ANOVA (C, D) *t*‐test (E–H), and was shown as mean ± SD. ns, no statistically significant; *P < 0.05, **P < 0.01, and ****P < 0.0001.

To further explore the impact of ASS1 on the biological behavior of BC, we transfected the siNC or siASS1 into shPGAM1 BC cell lines. First, we verified the knockdown efficiencies of siASS1 in shPGAM1‐BC cell lines by western blotting (Fig. 4A,B). We then conducted related functional experiments to compare the malignant biological behaviors between these BC cells. We showed that knockdown of *ASS1* could restore the proliferative ability of shPGAM1‐BC cells (Fig. 4C,D). In line with the proliferation‐suppressive role of ASS1, transwell and wound‐healing assays suggested that knockdown of ASS1 could promote the invasive (Fig. 4E,F) and migratory (Fig. 4G,H) abilities of shPGAM1‐BC cell lines. Moreover, as shown by results of the western blot (Fig. 4I,J), shPGAM1‐BC cell lines could regain the expressions of EMT‐related markers by the knockdown of *ASS1*. Compared to that in the shPGAM1‐BC cell lines transfected with siNC, the expression of E‐cadherin in shPGAM1‐BC cell lines transfected with siASS1 was downregulated, whereas the expressions of Snail, Slug, Vimentin, and N‐cadherin were upregulated. These results indicated that ASS1 is necessary for PGAM1‐mediated BC proliferation, invasion, migration, and EMT process *in vitro*.

### 3.5 *PGAM1* downregulates *ASS1* expression through *cAMP/AMPK/CEBPB* axis

**Fig. 5:**
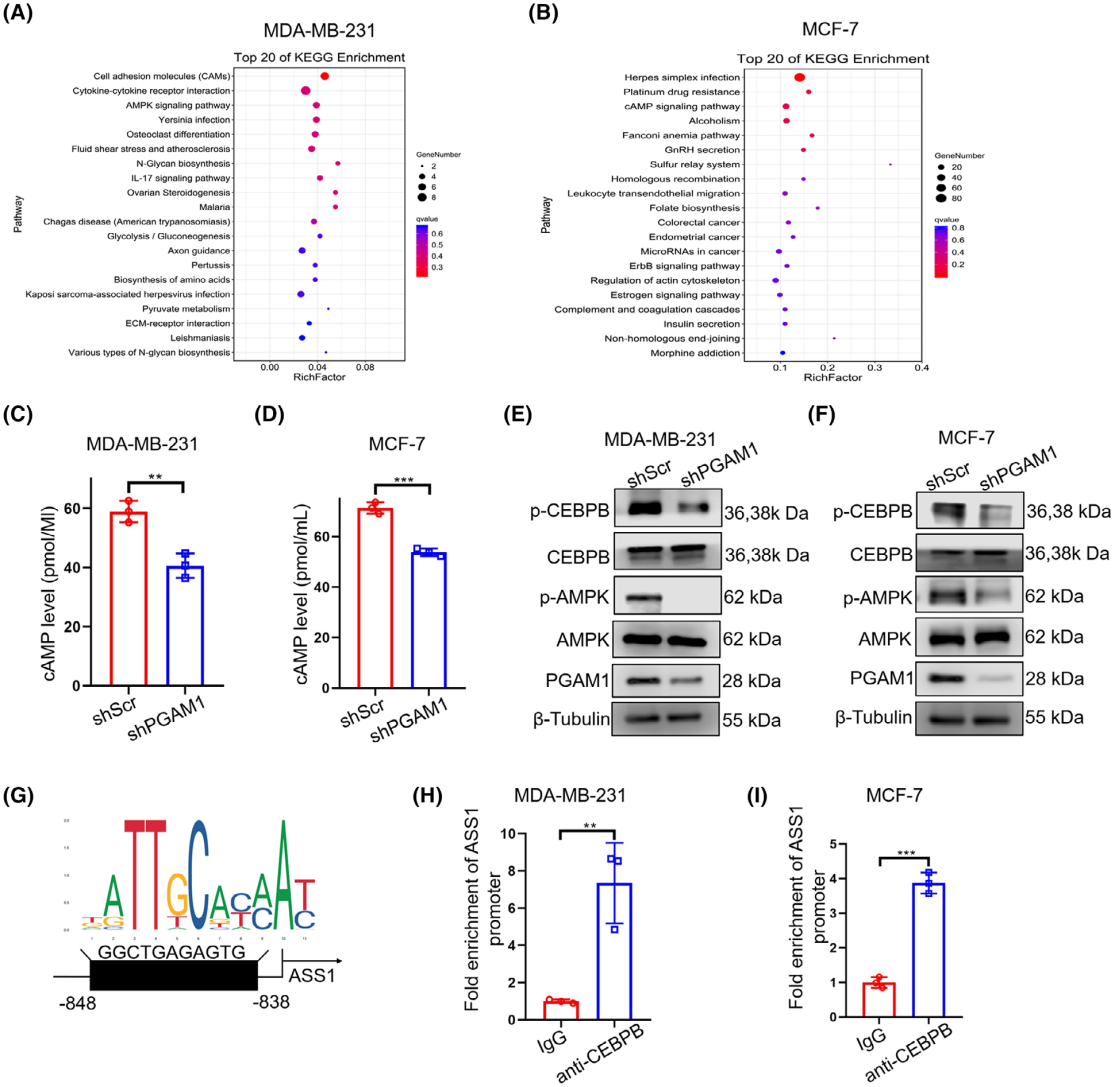
PGAM1 downregulates ASS1 expression through cAMP/AMPK/CEBPB axis. (A, B) KEGG analysis of PGAM1‐related downstream pathways. (C, D) The cAMP levels in the cell supernatants of the BC cell lines were detected by ELISA (*n* = 3). (E, F) The protein levels of AMPK, pAMPK, CEBPB, and pCEBPB measured by western blot (*n* = 3). (G) The JASPAR database was used for forecasting the binding sites of *CEBPB* in the *ASS1* promoter. (H, I) The ChIP assays were conducted to verify the actual binding site in BC cells (*n* = 3). (Data was analyzed by *t*‐test, and was shown as mean ± SD. **P < 0.01, ***P < 0.001.).

To dissect the molecular mechanism of PGAM1 regulation on ASS1 expression, we performed Kyoto Encyclopedia of Genes and Genomes (KEGG) analysis to explore *PGAM1*‐related downstream pathways through DEGs enrichment between shScr‐ and shPGAM1‐BC cells. We revealed that the cAMP/AMPK signaling pathway was closely related to PGAM1 in both MDA‐MB‐231 (Fig. 5A) and MCF‐7 cell lines (Fig. 5B), indicating that this signaling pathway may be involved in the oncogenic effects of PGAM1 in BC. We then confirmed that the cAMP levels (Fig. 5C,D) and phosphorylation level of AMPK (Fig. 5E,F) were markedly downregulated in *PGAM1*‐silencing BC cell lines, which was accordant with the evidence in KEGG analysis. To further determine the downstream transcription factor of cAMP/AMPK signaling pathway, we used GeneCards and Cistrome Data Browser to analyze the transcription factor which have the binding sites of *ASS1* and are, as well, related to the AMPK pathway [30]. *CEBPB* was screened for a high score and the western blot results further proved that the expression of pCEBPB was distinctly reduced in *PGAM1*‐knockdown BC cells (Fig. 5E,F, Fig. S1). As the JASPAR database predicted that *CEBPB* might potentially bind with the −848 to −838 bp region of the *ASS1* promoter (Fig. 5G), we performed the ChIP assay, and the results indicated an obvious enrichment of the promoter of *ASS1* by anti‐CEBPB antibody in MDA‐MB‐231 (Fig. 5H) and MCF‐7 cells (Fig. 5I), which provides preliminary evidence for supporting our prediction. For further confirmation, we used the aminoimidazole‐4‐carboxamide ribonucleoside (AICAR) as a pharmacological activator of AMPK to enhance AMPK phosphorylation in *PGAM1* knockdown cells and assess the expression of CEBPB and ASS1. The result showed that AICAR treatment was able to upregulate the expression of pCEBPB and downregulate the expression of ASS1 (Fig. S2a,b). To further confirm the CEBPB regulation of ASS1, we constructed the siNC‐ and siCEBPB‐BC cell lines to measure ASS1 protein level. As we expected, knockdown of *CEBPB* in *PGAM1* proficient BC cell could also increase the ASS1 expression (Fig. S2c,d). Collectively, we identified *CEBPB* as a new negative transcriptional regulator of *ASS1* and demonstrated that PGAM1 regulates ASS1 expression through cAMP/AMPK/CEBPB axis to promote BC progression.

### 3.6 *PGAM1* promotes the tumor growth of BC via altering *ASS1* expression *in vivo*

**Fig. 6:**
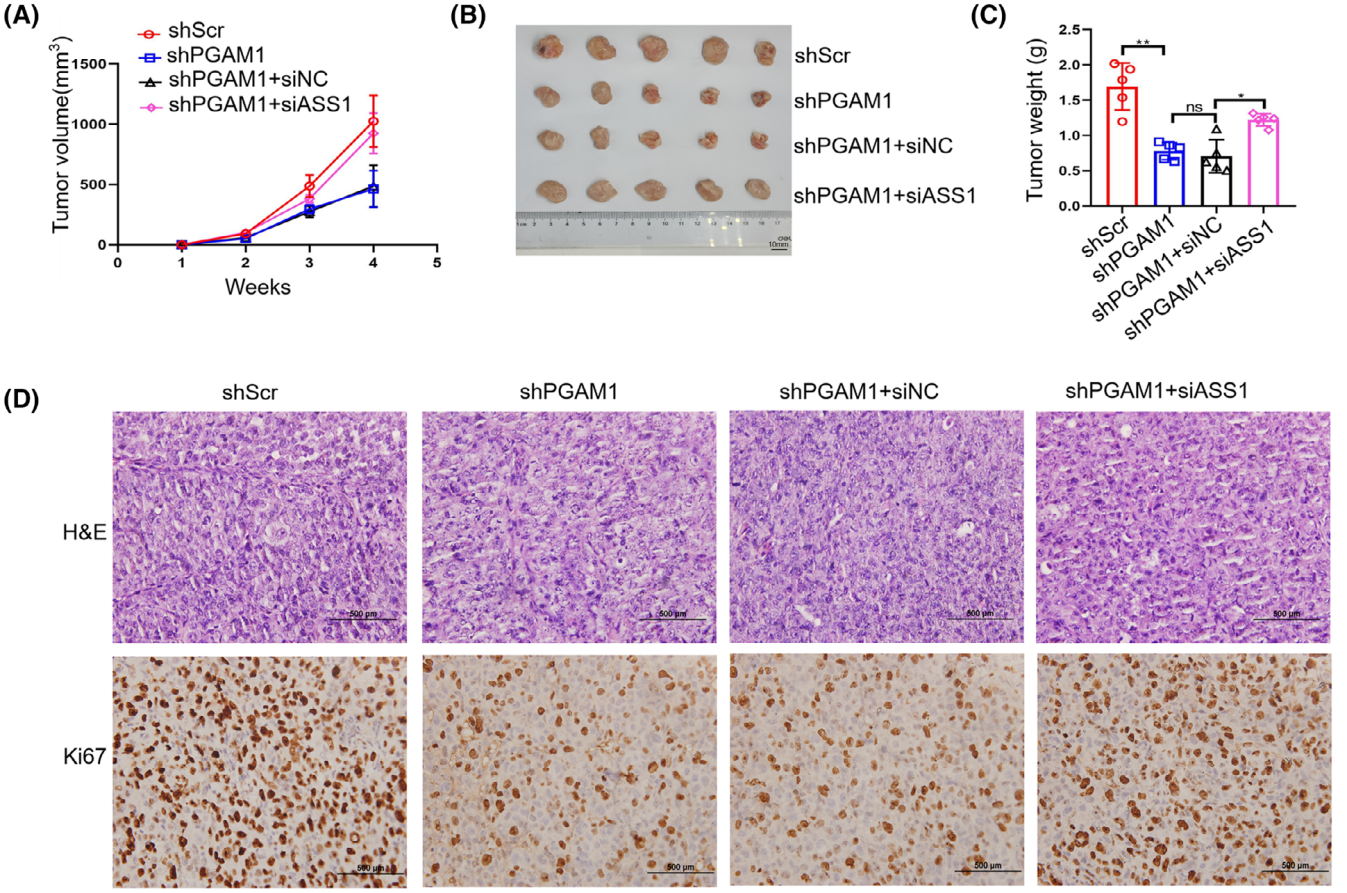
PGAM1 promotes the tumor growth of BC via altering ASS1 expression *in vivo*. (A) Tumor volumes measured each week (*n* = 5). (B) Tumors obtained from mice (*n* = 5, scale bar: 10 mm.) sacrificed on day 28 and (C) tumor weight data (*n* = 5). (D) Representative images of H&E and *Ki67* IHC staining of tumor tissues of tumor xenograft models (*n* = 5, scale bar: 500 μm). Data was analyzed by one‐way ANOVA (A) or *t*‐test (C), and was shown as mean ± SD. *P < 0.05, **P < 0.01.

To further verify the results of *in vitro* experiments showing that PGAM1 regulates the biological behavior of BC by affecting the expression of ASS1, xenograft models were constructed by injecting MDA‐MB‐231 cells into BALB/c nude mice. The tumor volumes and weights in the shPGAM1 group were distinctly reduced compared to that of the shScr group (Fig. 6A–C). Higher tumor volume and weight were measured in the shPGAM1+siASS1 group than that of shPGAM1+siNC group (Fig. 6A–C). H&E and proliferating cell‐associated antigen Ki67 staining were conducted to assess the proliferation abilities of tumors. We found that the nuclei in the shScr and shPGAM1+siASS1 groups were large and deeply stained, while the Ki67‐positive rate was significantly lower in the shPGAM1 and shPGAM1+siNC groups (Fig. 6D). Moreover, we assessed the expression of PGAM1 and ASS1 in tumor tissues of these four groups collected at the end of the experiment by IHC. The results showed that PGAM1 expression was lower in the shPGAM1 group than in the siNC group (Fig. S3a,b). Similarly, compared to shPGAM1+siNC group, ASS1 expression was relatively decreased in the shPGAM1+siASS1 group (Fig. S3c,d). Taken together, these data indicate that PGAM1 can promote tumorigenicity of BC *in vivo* by altering ASS1 expression.

### 3.7 Downregulated *ASS1* levels are associated with high *PGAM1* expression and worse prognosis in patients with BC

**Fig. 7:**
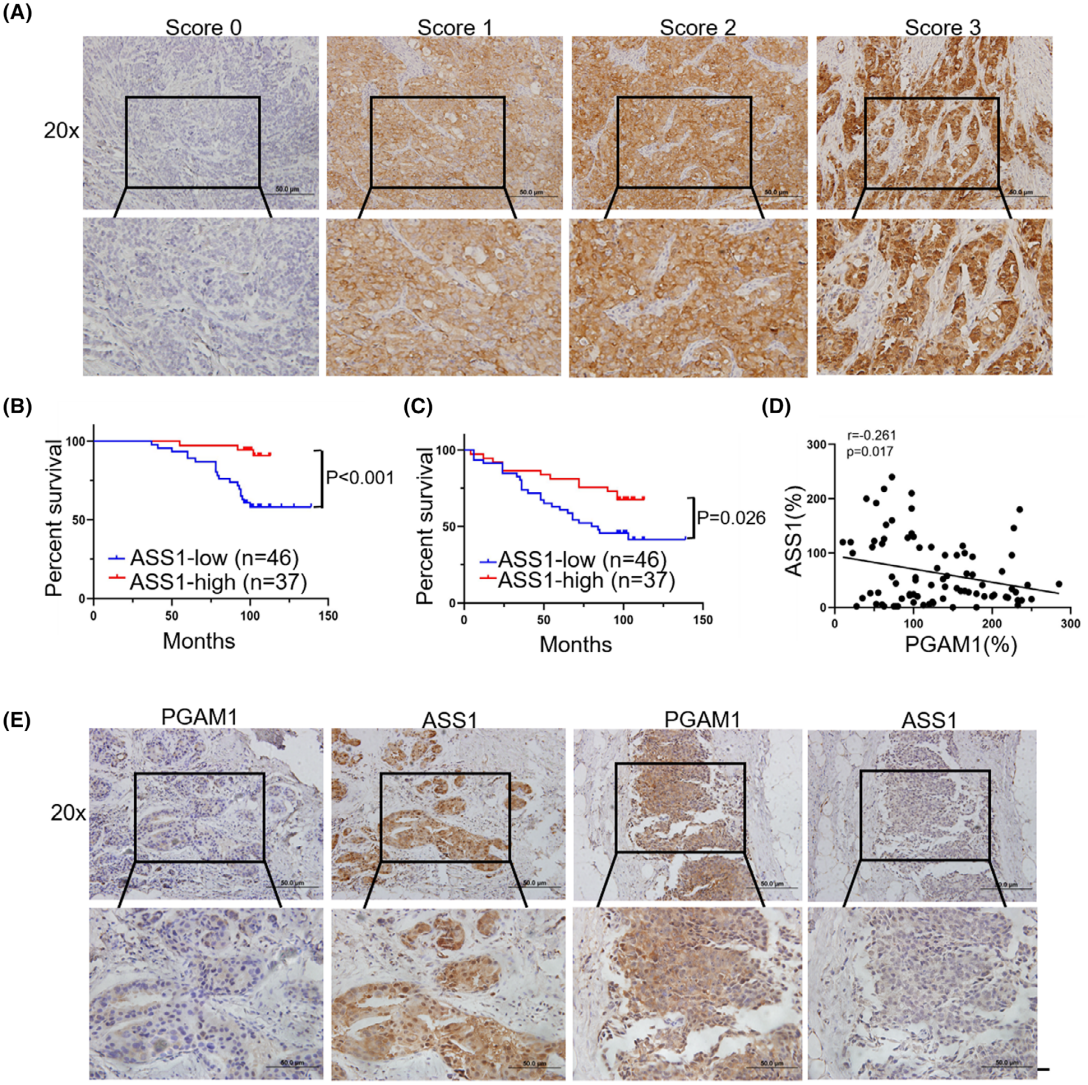
Downregulated ASS1 levels are associated with high PGAM1 expression and worse prognosis in patients with BC. (A) Representative images of ASS1 with different IHC staining scores (*n* = 83, scale bar: 50 μm). (B) Kaplan–Meier analysis of OS and (C) DFS in BC patients with low and high ASS1 expression levels (*n* = 83). (D) The expression levels of PGAM1 were negatively related to the expression levels of ASS1 (*n* = 83). (E) Representative images of IHC staining of the same regions for PGAM1 and ASS1 (*n* = 83, scale bar: 50 μm). Data was analyzed by Kaplan–Meier method (B, C) or Spearman's correlation (D).

We next identified the relationship between ASS1 levels and the patients' prognosis with BC. As shown in Fig. 7A, the score of ASS1 expression was classified into 0–3 based on the staining intensity of specimens. Immunohistochemistry (IHC) results validated that the low level of ASS1 was correlated with worse OS and DFS of patients with BC compared to high levels of ASS1 (Fig. 7B,C). Besides, the correlation between the expression levels of ASS1 and the clinical pathological features of patients was not significant, including age; LN metastasis; TNM stage; and ER, PR, and HER2 statuses (Table 1). Univariate analysis revealed that high expression levels of PGAM1 and TNM stage were high‐risk factors of OS, while the high expression level of ASS1 and ER positivity showed a protective effect (Table 2). Multivariate analysis demonstrated that the highly expressed PGAM1 was an independent high‐risk factor, and the highly expressed ASS1 was an independent protective effect (Table 2). Additionally, we found that the expression levels of ASS1 were negatively related to the expression levels of PGAM1 (Fig. 7D,E). Representative images of IHC in the same areas showed distinct expressions of PGAM1 and ASS1. Thus, the above mentioned data further supported the previous results that PGAM1/ASS1 contributes to the malignant behavior of BC.

**Table 2:** Cox proportional hazards models between clinical and survival in patients with breast cancer.

Variables	Univariable		Multivariable	
	HR (95% CI)	*P* value	HR (95% CI)	*P* value
PGAM1	6.530 (2.205–19.340)	0.000	6.192 (2.074–18.482)	0.001
ASS1	0.158 (0.047–0.535)	0.003	0.169 (0.049–0.575)	0.004
Age (≥ 50 years vs. < 50 years)	0.780 (0.337–1.806)	0.562		
LN metastasis (Yes vs. No)	2.442 (0.995–5.995)	0.051		
TNM stage (III + II vs. I)	3.461 (1.024–11.700)	0.046		
ER (Positive vs. Negative)	0.428 (0.185–0.988)	0.047		
PR (Positive vs. Negative)	0.630 (0.272–1.458)	0.280		
HER2 (Positive vs. Negative)	1.526 (0.652–3.572)	0.330		

## 4 Discussion

PGAM1 is a pivotal enzyme closely related to glycolysis and biosynthesis [31]. We previously demonstrated that the mTOR signaling pathway stimulates PGAM1 through the upregulation of *HIF‐1α*. *PGAM1* knockdown suppresses glycolysis and cancer genesis due to oncogenic mTOR signaling [8]. Here, we revealed that silencing of *PGAM1* could reconstruct the expression of *ASS1* in BC cells to exert tumor‐suppressive effects. We proposed a novel molecular mechanism by which *ASS1* acts as the downstream effector molecule of *PGAM1* to regulate the malignant behavior of tumors, contributing to a more complete understanding of the PGAM1‐mediated molecular network in human tumors, and providing important implications for combination therapy.

Previous evidence has shown that the expression levels of PGAM1 are related to prognosis in many types of cancers [32]. Consistently, we confirmed here that elevated expression of PGAM1 was related to worse prognosis in publicly available datasets and tumor samples of BC. Other studies also indicated that knockdown of *PGAM1* could decrease the proliferative, invasive, and migratory abilities of tumor cells in pancreatic ductal adenocarcinoma [33] and urothelial bladder cancer [5]. Consistent with the above results, we found that PGAM1 promotes the malignant biological behaviors of BC cells *in vitro* and accelerates BC xenograft tumorigenesis *in vivo*. Additionally, it is well known that the EMT process is obviously related to malignant progression, and the mesenchymal characteristics induced by EMT prompt tumor cells to participate in the cascade of invasion and metastasis [34]. In NSCLC, EMT‐related protein expressions were markedly enriched in the highly expressed PGAM1 phenotype, indicating that PGAM1 may conduce to the EMT process [6]. Indeed, our results found that silencing of *PGAM1* distinctly downregulated the expression levels of mesenchymal markers and upregulated the expression levels of epithelial markers in BC cell lines. These findings suggested that PGAM1 plays a critical role in tumorigenesis and may act as a novel diagnostic and prognostic biomarker for BC patients.

A recent study documented that PGAM1 is activated by miR‐3614‐5p and serves a pro‐tumor role in tumor progression via regulation of transforming growth factor‐β signaling in NSCLC [6]. Another research in BC showed that PGAM1 directly binds to α‐smooth muscle actin gene to mediate the assembly, movement, and metastatic potential of tumor cells in a metabolically independent manner [35]. Moreover, in osteosarcoma, the sphingosine 1‐phosphate (S1P)/S1P3 receptor axis enhances the transcription of *PGAM1* and accelerates tumor cell growth [36]. Collectively, these data suggest that the molecular network mediated by PGAM1 is complex and essential for tumor development. Therefore, we used RNA sequencing to determine ASS1 acting as the downstream target of PGAM1, and it was highly expressed in *PGAM1*‐silenced BC cells. Then, we utilized co‐IP assays to verify that PGAM1 and ASS1 could interact with each other to exert their biological effects. Knockdown of *PGAM1* suppresses proliferation, invasion, migration, and EMT process in BC cells, indicating that ASS1 is a key downstream effector molecule of PGAM1, and participates in the regulation of malignant transformation.

ASS1 catalyzes the conversion of aspartic acid and citrulline into argininosuccinate that is cleaved by argininosuccinate lyase to produce arginine [37]. Arginine is a semi‐essential amino acid related to the synthesis of various metabolites affecting cancer cell growth, invasion, and metastasis [38]. Patients with HCC cannot synthesize arginine *de novo* due to the lack or low expression of ASS1, and are extremely dependent on exogenous arginine to maintain the necessary biological activities [18]. Therefore, ASS1 presents a strictly arginine‐dependent phenomenon in HCC. Studies documented that overexpression of ASS1 can markedly suppress the metastatic potential of liver tumor cells *in vitro* and the rate of lung metastasis *in vivo* [14]. Consistently, knocking out *ASS1* could improve the invasion ability of liver tumor cells *in vitro* and *in vivo*. Indeed, our study validated that the high expression of ASS1 caused by the knockdown of *PGAM1* reduced the proliferation of tumor cells *in vitro*, and alleviated tumor growth in xenograft models. In accordance, silencing of *ASS1* in BC could restore tumor growth *in vitro* and *in vivo*. Moreover, we indicated that loss of ASS1 expression has obvious prognostic value for poor prognosis in BC, which is consistent with other studies in HCC, glioblastoma, and several other cancers [39, 40]. However, a previous study reported that ASS1 is highly expressed and is positively related to the aggressiveness and worse prognosis of gastric cancer [41]. ASS1 is also highly expressed in colorectal tumors and can be used as a target in human primary colorectal tumors [42]. The application of ASS1 inhibitors or *ASS1* knockout weakens the pathogenicity of colorectal tumors [42]. Thus, the relation of prognosis to ASS1 in different types of tumors may be different from that in BC patients, which should be carefully considered. The double‐faced function of ASS1 in distinct tumors is still controversial, and further research is required to explore the underlying mechanisms. Moreover, the effect of *PGAM1* knockdown on arginine production in BC cells needs to be clarified in future studies.

cAMP/AMPK signaling was a common altered pathway in tumors. As a major downstream transcription factor of AMPK pathway, *CEBPB* is reported to be required for, or facilitates the development of various types of tumors [43]. For example, *CEBPB* ^−/−^ mice are completely resistant to papilloma induced by chemical carcinogens [44], which was in accord with our report that silencing of *CEBPB* can increase *ASS1* expression and alleviate the malignant biological behaviors and tumor progression. Indeed, *CEBPB* was identified to directly bind to the promoter of cytoplasmic polyadenylation element‐binding protein 1, and thus, could inhibit transcription [45]. Veremeyko et al. [46] showed that CEBPB is highly expressed in M1 macrophages, and it appears to inhibit *Egr2*, leading to loss of plasticity. Similarly, we believe that *CEBPB* is a negative transcriptional regulator of *ASS1*. Here, we showed that PGAM1 can upregulate cAMP levels and then activate the AMPK signaling pathway, altering the phosphorylation level of CEBPB, thereby affecting its transcriptional activity and combination with ASS1, which suppress the ASS1 expression and contribute to BC progression.

Additionally, our data have also indicated an interaction between PGAM1 and ASS1. In the view that ASS1 is a key enzyme of arginine biological process, it is possible that the interaction between PGAM1 and ASS1 was present in order to regulate glycolytic flux to support cellular arginine synthesis, which indicated a close connection between glucose metabolism and amino acid metabolism, contributing to playing important roles in tumorigenesis. However, it is obvious that more broad explorations needed to be verified. Accordingly, to understand the physiological or pathological significance of interactions between these metabolic enzymes becomes very important.

In summary, our research aims to deepen our understanding on the PGAM1‐mediated ASS1 expression contributing to tumorigenesis and progression, and to uncover potential targets for combined therapy strategies in BC. Importantly, most treatment strategies focus on the effect of arginine starvation while neglecting the tumor suppressor effect of ASS1 [47]. Our study indicated for the first time that knockdown of *PGAM1* could reconstruct the expression of ASS1 in BC cells, resulting in a decrease in tumor growth and exerting antitumor effects via cAMP/AMPK/CEBPB axis. These findings constructed a new theoretical background for PGAM1‐mediated tumor progression, and the elements in this molecular network could be the target of BC treatment.

## 5 Conclusion

PGAM1 and ASS1 are important enzymes in tumor metabolism. In this study, we investigated the biological effect of PGAM1 and ASS1 on patients' prognosis in BC. Interestingly, we demonstrated that PGAM1 negatively regulates ASS1 expression through the cAMP/AMPK/CEBPB axis. Collectively, our data highlight a greater understanding of PGAM1‐mediated molecular network and provide potential targets and combination therapeutic strategies in BC.

## Conflict of interest

The authors declare no conflict of interest.

## Author contributions

ML and QS conceived and designed the experiments. ML, RL, MW, TL, QZ, and DZ performed the experiments. JW, MS, and XR provided some suggestions. Data analysis was performed by ML and QS. Writing, reviewing, and manuscript editing was done by ML, XR and QS. All authors read and approved the final paper.

## Peer review

The peer review history for this article is available at https://publons.com/publon/10.1002/1878-0261.13259.

## Supporting information


**Fig. S1.** Knockdown of *PGAM1* increases *ASS1* expression, decreases *AMPK/CEBPB* expression and inhibits BC cell proliferation, invasion, migration, and EMT process *in vitro*.

**Fig. S2.** *ASS1* expression was negatively regulated by *AMPK/CEBPB* signal pathway.

**Fig. S3.** The expression level of *PGAM1* and *ASS1* in tumor tissue of xenograft model.

**Table S1.** Antibodies used for immunohistochemical (IHC), western blotting (WB) and Co‐Immunoprecipitation (Co‐IP).

**Table S2.** siRNA and shRNA sequence used in the present study.

**Table S3.** Sequences of primers used for qRT‐PCR.

## Data Availability

The study relevant data are presented in the manuscript. Requests for data, experiment protocols, or any other questions can be made addressed to sunqian923@126.com.
