# Supplementary figures and images for: PGAM1 regulation of ASS1 contributes to the progression of breast cancer through the cAMP/AMPK/CEBPB pathway
Source: Mol Oncol. 2022 Jun 27;16(15):2843–60. doi: 10.1002/1878-0261.13259 (PMC9348593; doi:10.1002/1878-0261.13259)

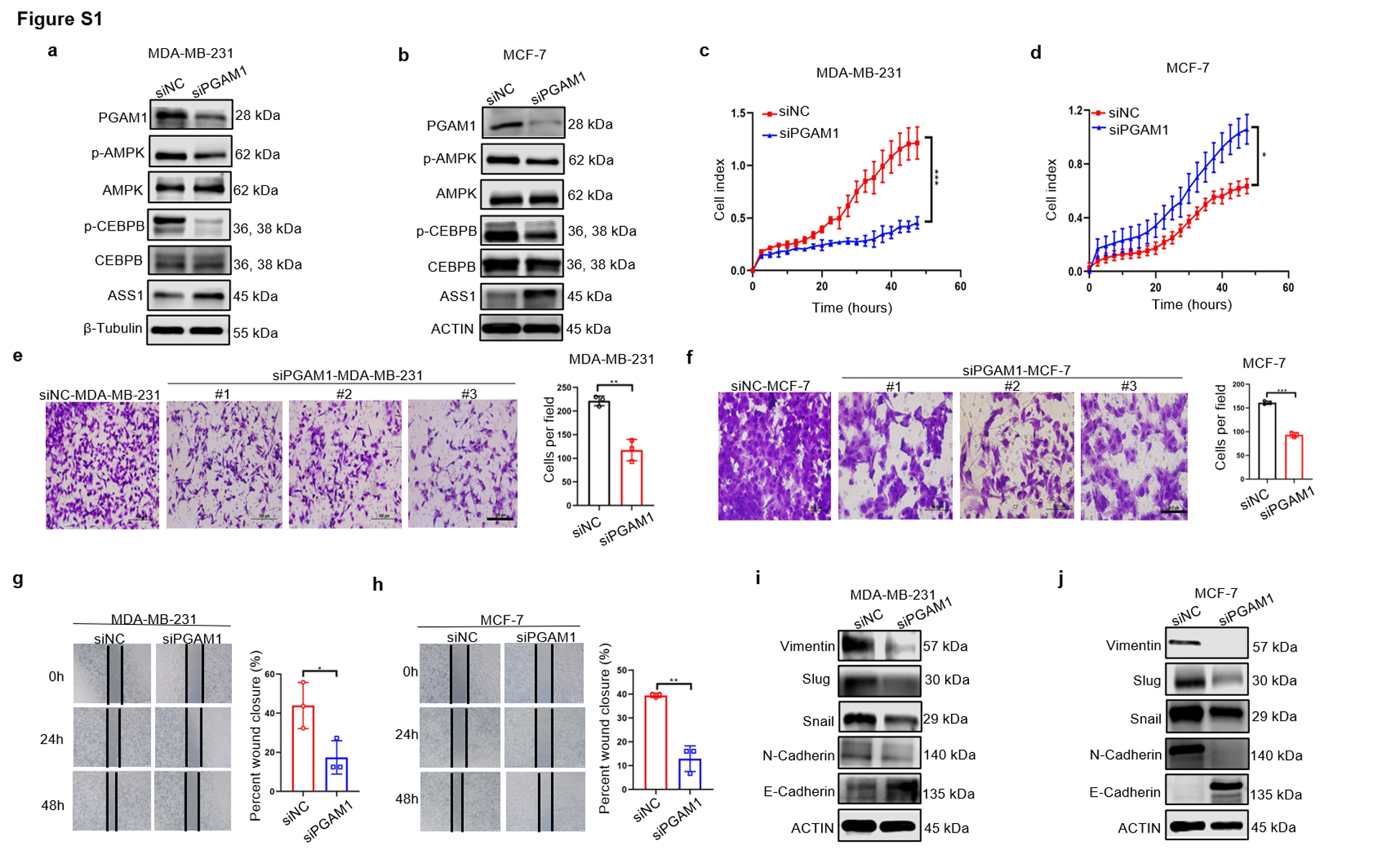

Supplement: Supplementary file 1 — Fig. S1. Knockdown of PGAM1 increases ASS1 expression, decreases AMPK/CEBPB expression and inhibits BC cell proliferation, invasion, migration, and EMT process in vitro. [file MOL2-16-2843-s006.tif]

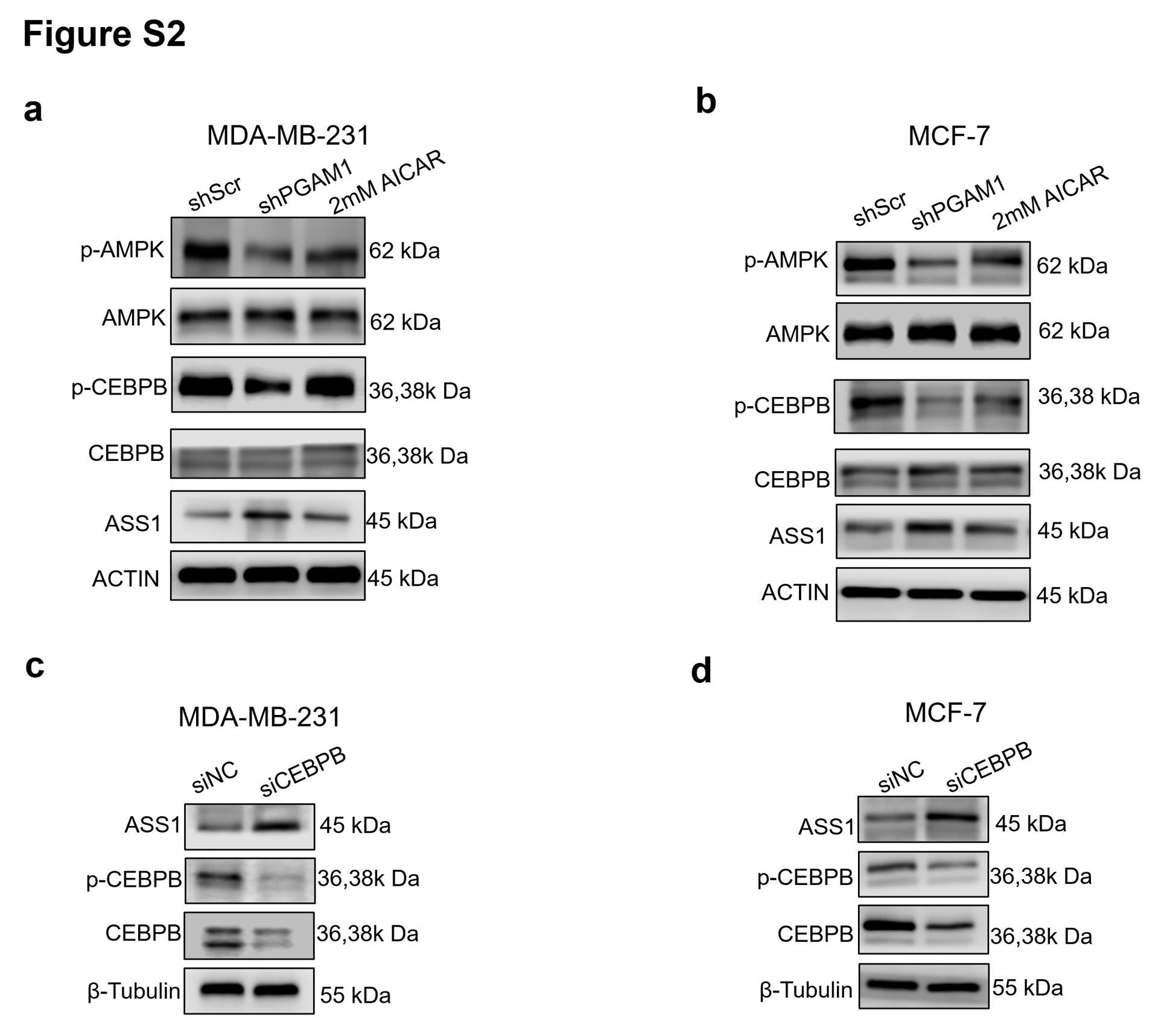

Supplement: Supplementary file 2 — Fig. S2. ASS1 expression was negatively regulated by AMPK/CEBPB signal pathway. [file MOL2-16-2843-s004.tif]

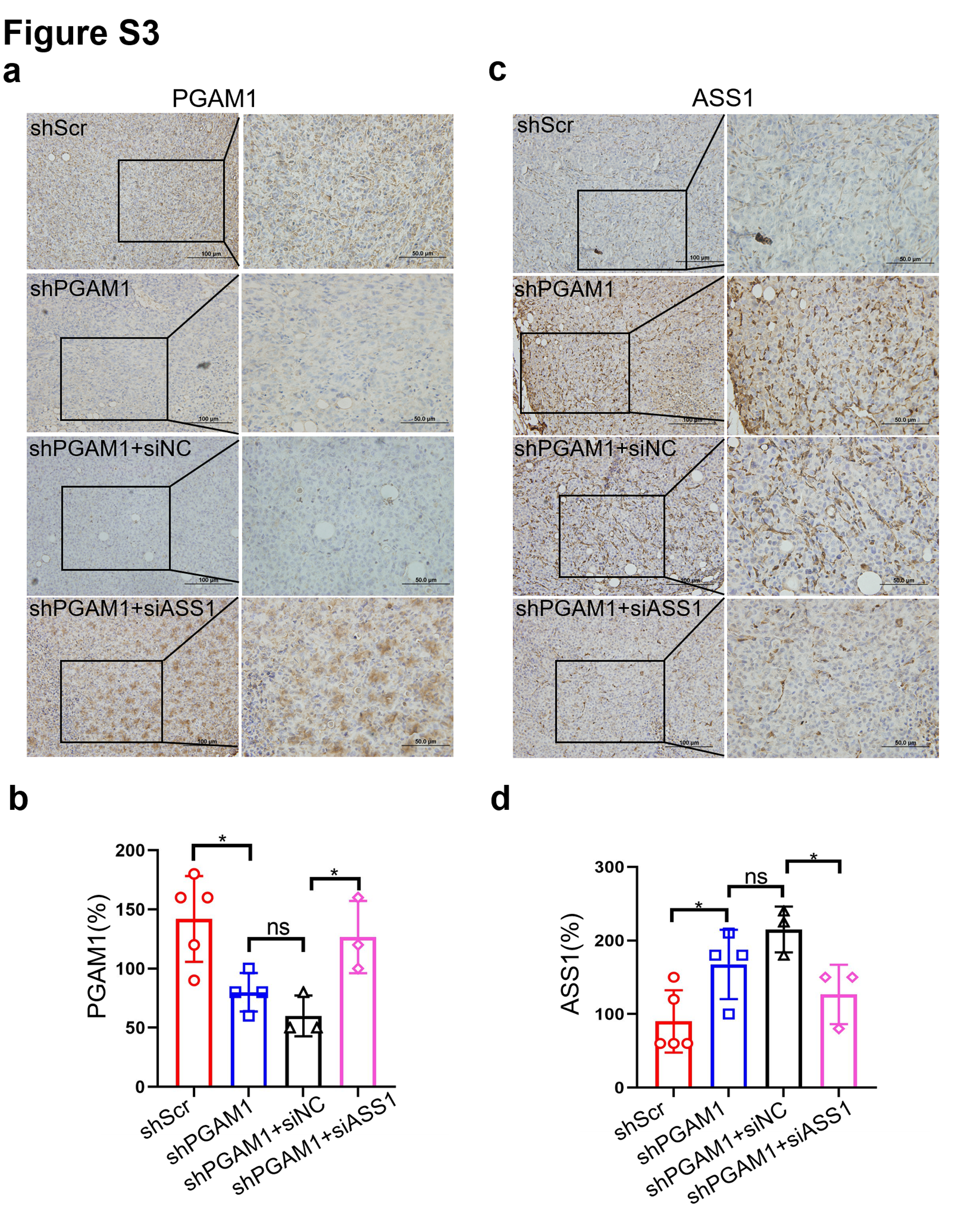

Supplement: Supplementary file 3 — Fig. S3. The expression level of PGAM1 and ASS1 in tumor tissue of xenograft model. [file MOL2-16-2843-s002.tif]
